# Temporal Tampering Detection in Automotive Dashcam Videos via Multi-Feature Forensic Analysis and a 1D Convolutional Neural Network

**DOI:** 10.3390/s26020517

**Published:** 2026-01-13

**Authors:** Ali Rehman Shinwari, Uswah Binti Khairuddin, Mohamad Fadzli Bin Haniff

**Affiliations:** 1 Malaysia-Japan International Institute of Technology, Universiti Teknologi Malaysia, Kuala Lumpur 54100, Malaysia; uswah.kl@utm.my (U.B.K.); mfadzlihaniff@utm.my (M.F.B.H.); 2 Department of Computer Software and Security, American University of Kurdistan, Zakho Road Sumel, Duhok 42001, Iraq

**Keywords:** video tampering detection, automotive dashcam forensics, 1D convolutional neural network (CNN), multi-feature temporal analysis, frame insertion, deletion, duplication

## Abstract

**Highlights:**

- Lightweight multi-feature temporal forensic framework that fuses frame-difference magnitude, SSIM drift, optical-flow mean, forward–backward flow error, and temporal prediction error, modeled with a shallow 1D-CNN for dashcam video tampering detection.
- Near real-time CPU inference (≈12.7–12.9 FPS) with minimal memory overhead (≈0.085 MB average), enabling practical deployment on embedded and forensic systems without GPUs.
- Strong intra-dataset performance on D^2^-City: 95.0% accuracy for frame deletion, 100.0% for insertion, and 95.0% for duplication; multiclass detection achieves 96.3% accuracy and class-wise AUCs up to 1.0.
- Cross-dataset analysis reveals domain shift, with insertion remaining robust (up to ≈97% accuracy on VIRAT), while deletion/duplication degrades on VIRAT and BDDA, motivating domain adaptation strategies.
- Ablation shows depth and temporal receptive field matter: two Conv1D blocks and kernels of 5–7 outperform shallower/smaller setups without meaningful efficiency penalties.

**Abstract:**

Automotive dashboard cameras are widely used to record driving events and often serve as critical evidence in accident investigations and insurance claims. However, the availability of free and low-cost editing tools has increased the risk of video tampering, underscoring the need for reliable methods to verify video authenticity. Temporal tampering typically involves manipulating frame order through insertion, deletion, or duplication. This paper proposes a computationally efficient framework that transforms high-dimensional video into compact one-dimensional temporal signals and learns tampering patterns using a shallow one-dimensional convolutional neural network (1D-CNN). Five complementary features are extracted between consecutive frames: frame-difference magnitude, structural similarity drift (SSIM drift), optical-flow mean, forward–backward optical-flow consistency error, and compression-aware temporal prediction error. Per-video robust normalization is applied to emphasize intra-video anomalies. Experiments on a custom dataset derived from D^2^-City demonstrate strong detection performance in single-attack settings: 95.0% accuracy for frame deletion, 100.0% for frame insertion, and 95.0% for frame duplication. In a four-class setting (non-tampered, insertion, deletion, duplication), the model achieves 96.3% accuracy, with AUCs of 0.994, 1.000, 0.997, and 0.988, respectively. Efficiency analysis confirms near real-time CPU inference (≈12.7–12.9 FPS) with minimal memory overhead. Cross-dataset tests on BDDA and VIRAT reveal domain-shift sensitivity, particularly for deletion and duplication, highlighting the need for domain adaptation and augmentation. Overall, the proposed multi-feature 1D-CNN provides a practical, interpretable, and resource-aware solution for temporal tampering detection in dashcam videos, supporting trustworthy video forensics in IoT-enabled transportation systems.

## 1. Introduction

Automotive dashboard cameras (dashcams) have become increasingly common in modern vehicles, offering valuable evidence for accident investigations, insurance claims, and law enforcement. Despite their benefits, the widespread availability of inexpensive video editing tools has raised concerns about video authenticity. Courts and insurance companies require trusted forensic reports to validate video evidence; this makes tampering detection a critical cybersecurity challenge in IoT-enabled transportation systems [1]. Video tampering can occur in the temporal domain (frame insertion, deletion, duplication), the spatial domain (object removal or modification), or both (spatio-temporal tampering) [2]. Temporal tampering is particularly challenging because it alters the sequence of frames without obvious visual artifacts, making detection difficult in dynamic driving environments.

Several approaches have been proposed to address this problem. Early research primarily relied on handcrafted features and statistical inconsistencies. For example, inconsistencies in correlation coefficients were used to detect insertions and deletions [3], while extensions incorporating Local Binary Pattern descriptors improved robustness under varying conditions [4]. Other studies analyzed prediction residuals, optical flow gradients, and compressed-domain features [5], or proposed descriptors such as Pseudo Flow Orientation Variation (PFOV) combined with robust principal component analysis [6]. Additional works used time–frequency analysis of reconstructed DCT coefficients [7], motion vector inconsistencies [8], entropy-based descriptors [9], and Haralick feature correlations for detecting insertion, deletion, and duplication events [10]. Although these handcrafted methods demonstrate promising results, many are sensitive to moving background and compression.

Among motion-based features, optical flow has been widely investigated for temporal tampering detection. Optical flow represents the apparent motion of intensity patterns, edges, and surface structures between consecutive video frames and provides a direct characterization of temporal motion continuity. In non-tampered videos, optical flow variations across adjacent frames typically exhibit smooth and consistent behavior. In contrast, temporal manipulations such as frame insertion, deletion, or duplication introduce abrupt motion discontinuities, resulting in irregular fluctuations in optical flow magnitude and direction. These anomalies can be exploited as forensic cues to detect temporal tampering by identifying violations of natural motion consistency [11].

An optical flow–based approach for detecting and localizing video forgeries by analyzing disruptions in the optical flow sequence was proposed by [12]. Their method could detect frame addition, removal, and duplication events through motion inconsistency analysis. However, the approach was limited to videos captured with stationary cameras, which significantly restricts its applicability in real-world scenarios involving camera motion, such as automotive dashcam footage. Experimental results reported detection accuracy of 93.3% and 86.7% on two benchmark datasets.

Similarly, Ref. [13] developed a temporal tampering detection method based on optical flow consistency. Optical flow vectors in both horizontal and vertical directions were computed between neighboring frames, and consistency measures derived from these vectors were used as input features for a support vector machine classifier. For single-type tampering scenarios, the method achieved classification accuracies of 98.41%, 98.20%, 86.82%, and 92.61% in the x-direction for 25-frame insertion, 100-frame insertion, 25-frame deletion, and 100-frame deletion, respectively. Corresponding accuracies in the y-direction were 98.60%, 98.54%, 86.02%, and 88.56%. When both insertion and deletion attacks were considered jointly, the reported detection accuracies were 91.72% and 90.00% for 25-frame insertion and deletion, respectively, while accuracies of 89.83% and 92.63% were obtained for 100-frame insertion and deletion.

To detect copy-move forgery, structural similarity can be used. The method computes similarity scores between consecutive frames, leveraging the observation that duplicated frames exhibit significantly higher similarity compared to normal inter-frame sequences. A temporal similarity strategy applied to short subsequences enables effective detection of copy-move operations, while also facilitating precise localization of forged regions. Experimental evaluation on 15 videos captured under both stationary and dynamic conditions using digital and mobile cameras demonstrated exceptional performance, achieving a detection accuracy of 99.7%, surpassing prior approaches [14].

A passive video tampering detection method based on the consistency of the quotient of mean structural similarity (QoMSSIM) between adjacent frames. In original videos, QoMSSIM values remain relatively stable, whereas tampered videos—affected by frame insertion or deletion—exhibit noticeable disruptions in this consistency. The method involves computing MSSIM between adjacent frames, deriving QoMSSIM values, and applying post-processing and normalization to reduce the influence of video content variation. These processed values are then transformed into histogram features, which serve as input to a support vector machine (SVM) classifier. Experimental results on a large video dataset demonstrated high classification accuracy: 95.7% for single-type tampering and 92.27% and 92.75% for combined 25-frame and 100-frame insertions and deletions, respectively. The method also showed robustness against recompression and white Gaussian noise [15].

A passive video tampering detection method that leverages temporal signatures introduced by MPEG compression has been proposed by [16]. The approach reconstructs the video using frame prediction and compares temporal differences between adjacent frames of the original and reconstructed sequences. Significant deviations in prediction error indicate manipulations such as frame insertion, deletion, or duplication. To enhance reliability, the method incorporates optical flow analysis for validation and localization of tampered regions. Experimental results on videos with both fixed and adaptive GOP structures achieved an average detection accuracy of 87.5%, demonstrating robustness across diverse compression schemes.

With the introduction of deep learning, more powerful CNN-based and hybrid architectures emerged. Examples include 2D CNNs combined with SVM classifiers [17], 3D CNNs operating on absolute frame differences [18], contrastive-learning-based frameworks capturing motion continuity [19], and hybrid CNN–LSTM or CNN–GRU models for long-range temporal modeling [20]. At the same time, lightweight supervised and unsupervised frameworks were investigated, such as histogram-similarity-based SVM detection [21], correlation-distance-based dual-threshold methods [22], statistical descriptors using Sobel edges [23], and comparisons between supervised CNNs and unsupervised VGG-based representations [24]. Additional approaches utilized machine learning classifiers [25], regression-based outlier detection [26], motion vector and transform features for frame shuffling attacks [27], SIFT- and RANSAC-based duplication localization [28], tensor-based frameworks [29], KPCA-reduced VGG-16 features [30], and CNN-based architectures such as VFID-Net [31]. Other studies explored motion-residual and object-tracking-based tampering detection [32], and ensemble-based tampering detectors using Haralick and LBP features have also been reported [33]. Although these works show significant progress, they often require heavy computation, rely on dataset with static background or with very little movements, none of them are trained and tested on automotive dashcam dataset.

Recent work has explored complementary strategies for multimedia security and forensics. Ref. [34] introduced a segment-level encryption framework leveraging temporal action segmentation and chaotic key generation, highlighting the importance of modeling temporal structural variations. Ref. [35] proposed a grid recovery method for JPEG recompression forensics using a learnable second-order difference layer and color fine-grain representation, emphasizing the role of structural and chrominance cues in detecting manipulation. These insights complement our multi-feature approach by suggesting future directions such as segment-level modeling and color-aware feature integration.

Despite these advancements, three major gaps remain: (1) most existing studies focus on static or surveillance datasets rather than real-world dashcam recordings; (2) many deep learning architectures are computationally expensive and unsuitable for large-scale deployment; and (3) few frameworks jointly address frame insertion, deletion, and duplication within a single unified model.

To address these gaps, this study proposes a computationally efficient approach for detecting temporal tampering in dashcam videos. The method extracts five complementary features—frame-difference magnitude, SSIM drift, optical flow mean, forward–backward flow error, and temporal prediction error—between consecutive frames and models them using a lightweight one-dimensional convolutional neural network (1D-CNN). These features are normalized by the video to emphasize intra-video anomalies. Experiments on a custom dataset derived from D^2^-City demonstrate strong detection performance in single-attack scenarios: 95.0% accuracy for frame deletion, 100.0% for frame insertion, and 95.0% for frame duplication. In a four-class setting (non-tampered, insertion, deletion, duplication), the model achieves 96.3% accuracy, with class-wise AUCs up to 1.0. These results highlight the potential of the proposed approach for near real-time deployment in forensic and automotive cybersecurity applications, strengthening trust in IoT-enabled transportation systems.

### Contribution

This study contributes the following:

- A lightweight multi-feature temporal forensic framework for detecting temporal tampering in automotive dashcam videos, integrating frame difference magnitude, structural similarity drift, optical flow consistency, and temporal prediction error features within a unified representation.
- A computationally efficient temporal modeling strategy that converts high-dimensional video data into compact one-dimensional feature sequences, enabling effective learning using a shallow 1D convolutional neural network (1D-CNN).
- A large-scale custom tampered dataset derived from the D^2^-City dashcam dataset, constructed using controlled frame deletion, insertion, and duplication operations under realistic compression and motion conditions to support reproducible forensic evaluation.
- A comprehensive ablation and feature contribution analysis, demonstrating the individual and combined impact of temporal forensic features on detection performance and model stability.
- An extensive computational efficiency evaluation, showing that the proposed method achieves near real-time inference on standard CPU hardware, making it suitable for resource-constrained forensic and embedded applications.
- Cross-dataset generalization experiments that highlight domain shift challenges in real-world dashcam videos and provide insights into the limitations and future directions of temporal tampering detection.

## 2. Materials and Methods

The proposed method prioritizes computational and memory efficiency by exploiting temporal inconsistencies between consecutive video frames. It integrates five complementary forensic features—frame-difference magnitude, structural similarity drift (SSIM drift), optical-flow mean, forward–backward flow consistency error, and compression-aware temporal prediction error—into a unified representation. These features collectively capture pixel-level changes, structural degradation, motion continuity, and prediction anomalies, providing a robust basis for detecting temporal tampering.

For each input video, frames are extracted sequentially and converted to grayscale to reduce complexity and eliminate color redundancy. All frames are resized to a fixed spatial resolution to ensure consistency across sources. Feature sequences are computed between consecutive frames and normalized per video to emphasize intra-video anomalies. By transforming high-dimensional video into compact one-dimensional temporal signals, the proposed approach substantially reduces memory usage and computational overhead, making it suitable for long-duration automotive dashcam videos.

**Figure 1:**
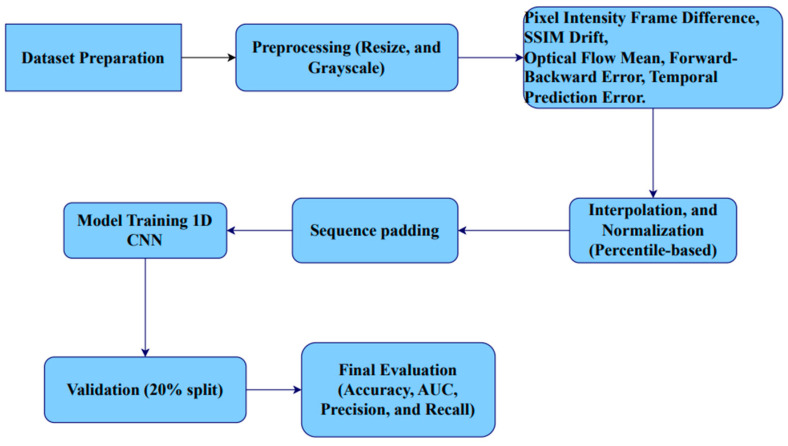
Workflow Diagram for Temporal Video Tampering Detection Using Multi Features Analysis and 1D-CNN.

Although frame-difference features form a strong baseline, real-world dashcam videos introduce challenges such as camera motion, illumination changes, and compression artifacts. To address these issues, the framework combines complementary features that capture structural similarity degradation, motion consistency, and prediction errors. These features are jointly modeled using a one-dimensional convolutional neural network (1D-CNN) to learn discriminative temporal patterns associated with tampering events. Figure 1 illustrates the overall workflow of the proposed method.

Let a video $V={\left\{I_{t}\right\}}_{t=1}^{T}$ consist of T consecutive grayscale frames captured by an automotive dashboard camera. Temporal tampering operations such as frame deletion, insertion, or duplication disrupt the natural temporal continuity of the video, while often leaving minimal perceptual artifacts in individual frames. The objective of this work is to determine whether a given video has undergone temporal tampering by analyzing temporal forensic inconsistencies rather than visual content.

The proposed framework formulates temporal tampering detection as a binary classification problem, distinguishing tampered and non-tampered videos using a compact set of lightweight temporal forensic features aggregated by a one-dimensional convolutional neural network (1D-CNN).

### 2.1. Overview of the Proposed Framework

The overall processing pipeline consists of four main stages:

- Preprocessing and frame standardization
- Temporal forensic features Extraction
- Feature sequence construction and normalization
- Model Architecture

### 2.2. Preprocessing and Frame Standardization

Each input video is decoded frame by frame using OpenCV version: 4.12.0. To reduce color redundancy and mitigate encoder-dependent chromatic variations, frames are converted to grayscale. All frames are resized to a fixed spatial resolution of 480 × 270 pixels, ensuring uniform feature scale and computational efficiency suitable for real-time dashcam scenarios.

Let $G_{t}$ denote the grayscale frame at time index t. For each consecutive frame pair $\left(G_{t-1},G_{t}\right)$, we compute five complementary temporal forensic features:

- **Frame Difference Magnitude**—sum of thresholded pixel-wise differences to capture abrupt intensity changes.
- **Structural Similarity Drift (SSIM Drift)**—inverse SSIM score to quantify structural degradation between frames.
- **Optical Flow Mean Magnitude**—average motion strength estimated via Farnebäck dense optical flow.
- **Forward–Backward Flow Consistency Error**—deviation between forward and backward flow fields to detect motion discontinuities.
- **Temporal Prediction Error**—deviation from a first-order temporal prediction model $G_{t}\approx 2G_{t-1}-G_{t-2}$, highlighting violations of natural motion continuity.

To ensure robustness, feature sequences are normalized per video using 2nd–98th percentile scaling and clipped to $\left[0,1\right]$, emphasizing intra-video anomalies while suppressing global variations.

### 2.3. Temporal Forensic Feature Extraction

For each pair of consecutive frames, a five-dimensional temporal feature vector is extracted. These features are intentionally designed to be lightweight, interpretable, and robust to compression, aligning with the constraints of automotive dashcam videos.

#### 2.3.1. Frame Difference Sum

**Figure 2:**
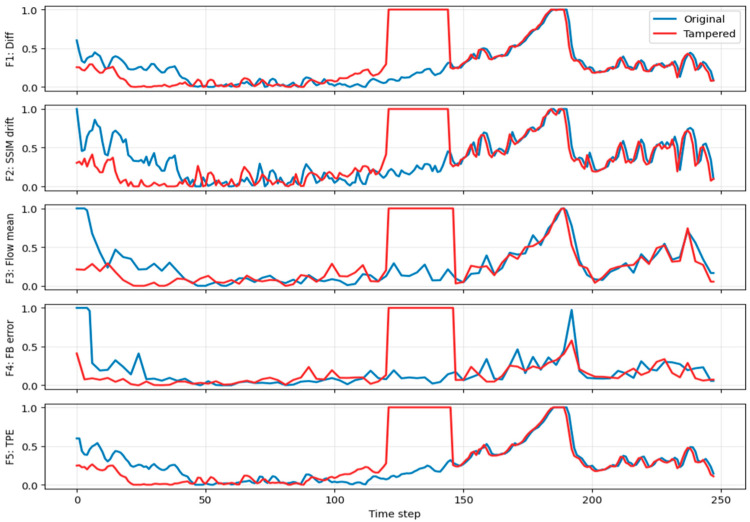
Frame Insertion.

**Figure 3:**
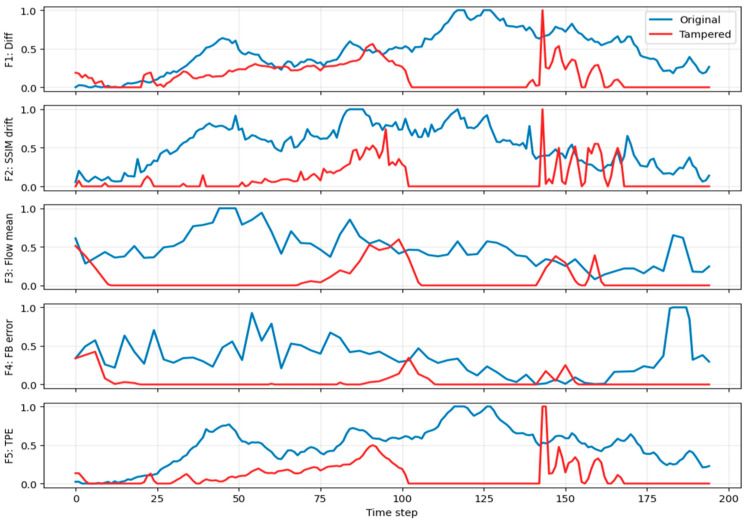
Frame Deletion.

**Figure 4:**
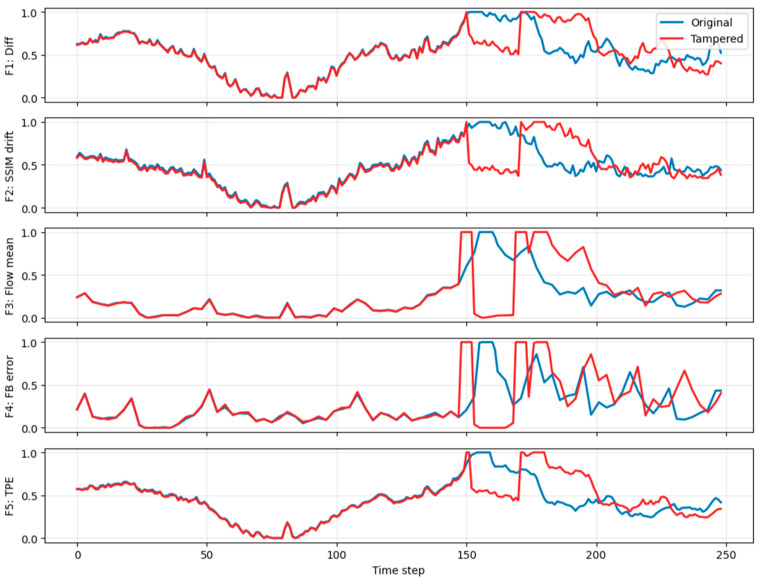
Frame Duplication.

Captures abrupt pixel-level changes between consecutive frames:

$$F_{1}(t)=\sum I\left(|G_{t}-G_{t-1}|>\tau \right)$$

where τ=30 suppresses minor illumination fluctuations while emphasizing structural discontinuities caused by insertion or deletion. Frame difference sum plotting for frame insertion, deletion, and duplication is shown in Figure 2, Figure 3 and Figure 4.

#### 2.3.2. Structural Similarity Index Measure Drift (SSIM Drift)

Measures structural degradation using the inverse SSIM index:

$$F_{2}\left(t\right)=1-SSIM(G_{t-1},\;G_{t})$$

This feature is robust to global brightness shifts and highlights temporal inconsistencies. Structural similarity drift plotting for frame insertion, deletion, and duplication is shown in Figure 2, Figure 3 and Figure 4.

#### 2.3.3. Optical Flow Mean Magnitude

Global motion strength is estimated using [36] dense optical flow. For computational efficiency, flow estimation is performed on half-resolution frames.

$$F_{3}(t)=\frac{1}{N}\sum ∥u_{t}(x,y)∥$$

where $u_{t}$ denotes the optical flow vector field and N is the number of pixels. This feature captures ego-motion dynamics and scene motion continuity typical in dashcam footage. Optical flow mean plotting for frame insertion, deletion, and duplication is shown in Figure 2, Figure 3 and Figure 4.

#### 2.3.4. Forward–Backward Optical Flow Consistency Error

Evaluates motion reversibility using forward–backward consistency:

$$F_{4}(t)=\frac{1}{N}\sum ∥u_{t}^{f}+u_{t}^{b}(x+u_{t}^{f})∥$$

Tampering disrupts this consistency, producing elevated errors. Forward-backward optical flow consistency error plotting for frame insertion, deletion, and duplication is shown in Figure 2, Figure 3 and Figure 4.

#### 2.3.5. Temporal Prediction Error

Quantifies deviations from a first-order prediction model:

$${\hat{G}}_{t}=2G_{t-1}-G_{t-2}$$

Sensitive to missing or duplicated frames that violate linear temporal evolution.

$$F_{5}(t)=\sum I\left(|G_{t}-{\hat{G}}_{t}|>\tau \right)$$

This feature is particularly sensitive to missing or duplicated frames that violate linear temporal evolution assumptions exploited by video encoders. Temporal prediction error for frame insertion, deletion, and duplication is shown in Figure 2, Figure 3 and Figure 4.

Figure 2 illustrates normalized feature trajectories for an original video (blue) and its tampered variant (red) containing an inserted frame block. The insertion introduces a pronounced plateau in F1 (frame-difference magnitude) and F2 (SSIM drift), as well as elevated values in F3 (optical-flow mean), F4 (forward–backward error), and F5 (temporal prediction error), confirming disruption of temporal continuity. Although the curves do not perfectly overlap outside the tampered region, this is expected due to per-video normalization (2nd–98th percentile) and timeline misalignment caused by insertion. These preprocessing steps are deliberate for robustness and do not affect model learning, which focuses on localized spikes near tampered intervals. Attribution analysis (Permutation Importance and Integrated Gradients) further confirms that the CNN prioritizes these regions rather than global offsets.

Figure 3 depicts feature trajectories for a deletion event where 25 consecutive frames were removed starting at frame 144. All five features—F1, F2, F3, F4, and F5—exhibit sharp, co-located deviations near the edit boundary (≈144). Specifically, F1, F2, and F5 show distinct spikes indicating abrupt temporal discontinuity; F4 increases around the splice due to motion inconsistency; and F3 changes trend as the motion trajectory re-phases after the gap. Outside the tampered region, blue/red curves diverge globally, which is expected because (i) features are normalized per video, so identical raw values can map to different normalized amplitudes; and (ii) deletion shifts the chronology of subsequent frames, making pointwise overlay without gap compensation produce global offsets. Diagnostics should therefore focus on localized divergence and spikes around the deletion rather than demanding perfect curve coincidence elsewhere.

Figure 4 demonstrates frame duplication tampering detected through multi-feature analysis. The tampered sequence exhibits a distinct anomaly between steps 149 and 171, corresponding to approximately 23 consecutive frames. Within this interval, F3 (optical flow mean) approaches zero, indicating negligible motion, which is characteristic of repeated frames. Sharp discontinuities at the segment boundaries are evident in F4 (forward–backward error) and F5 (temporal prediction error), confirming abrupt transitions caused by copy-paste operations. F2 (SSIM drift) forms a pronounced plateau across the same region, suggesting strong resemblance to an earlier portion of the video, likely near frame 22. Collectively, these indicators, low intra-block motion, high boundary inconsistencies, and elevated similarity drift, provide strong evidence of frame duplication tampering, where a block of frames was copied from an earlier segment and inserted later in the sequence.

Across all three tampering scenarios—insertion, deletion, and duplication—the normalized temporal feature trajectories reveal consistent diagnostic patterns that localize edits despite global misalignment. Insertion events produce sustained plateaus and elevated feature values within the inserted block, whereas deletion introduces sharp, co-located spikes at splice boundaries followed by global divergence due to timeline compression. Duplication exhibits near-zero optical flow inside the repeated segment and pronounced boundary discontinuities, coupled with similarity drift indicating content reuse. These localized anomalies across F1–F5 confirm that multi-feature analysis effectively captures temporal inconsistencies introduced by diverse tampering operations, enabling robust detection even under per-video normalization and chronology shifts.

All five temporal features, frame-difference magnitude (F1), SSIM drift (F2), optical-flow mean (F3), forward–backward flow error (F4), and temporal prediction error (F5), were retained to ensure robustness and generalization across diverse tampering scenarios. While certain features exhibit stronger responses for specific edit types (e.g., F1 and F2 for insertion, F3 for duplication), others provide complementary signals that become critical under different conditions such as mixed manipulations, compression artifacts, or subtle edits. Eliminating features based on a limited set of examples risks reducing detection sensitivity in real-world deployments, where tampering patterns may vary significantly. Retaining the full feature set allows the model to learn discriminative combinations and adapt to unseen cases, while feature importance analysis in future large-scale evaluations can guide potential pruning or fusion strategies.

### 2.4. Feature Sequence Construction and Normalization

For each video, the extracted features form a temporal feature matrix:

$$F\in R^{(T-1)\times 5}$$

where T is the number of frames. To ensure robustness and prevent dataset-level information leakage, normalization is applied independently to each feature dimension on a per-video basis using the 2nd and 98th percentiles. This approach emphasizes intra-video anomalies rather than absolute feature magnitudes across different videos. Missing values caused by stride-based optical flow computation are handled through linear interpolation to maintain sequence continuity. Missing values caused by stride-based optical flow computation are handled through linear interpolation to maintain sequence continuity.

### 2.5. Model Architecture

The proposed 1D Convolutional Neural Network (1D-CNN) is designed to model temporal dependencies in compact forensic feature sequences extracted from dashcam videos. Each video is represented as a variable-length sequence of five normalized temporal features, including frame difference magnitude, SSIM drift, optical flow mean magnitude, forward–backward optical flow error, and temporal prediction error. To enable batch processing, sequences are zero-padded to the maximum length observed in the training set.

The network applies one-dimensional convolutional filters along the temporal axis to capture local temporal patterns indicative of tampering events, such as motion discontinuities and prediction inconsistencies. The architecture consists of two convolutional blocks, each comprising a Conv1D layer with ReLU activation followed by max-pooling for temporal down sampling. The extracted features are flattened and passed to a fully connected layer with dropout regularization to mitigate overfitting. A sigmoid-activated output layer produces the final tampering probability.

**Figure 5:**
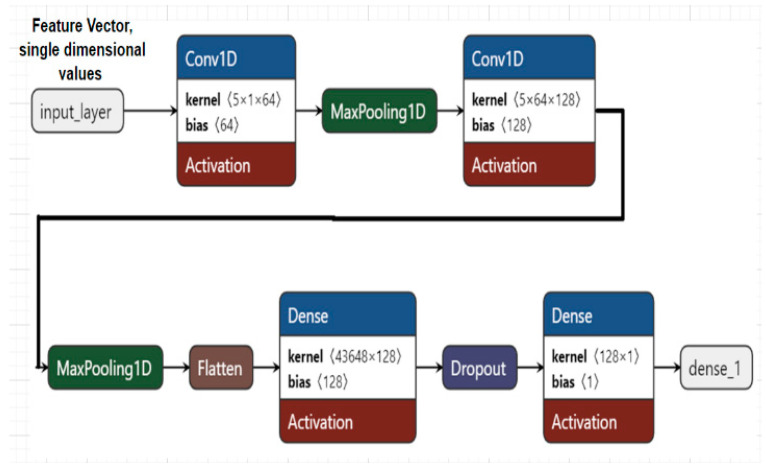
Model architecture.

The model is trained using the Adam optimizer with a learning rate of 1 × 10^−4^ and binary cross-entropy loss, reflecting the binary classification setting of tampered versus non-tampered videos. Figure 5 illustrates the overall architecture.

The architecture consists of two convolutional blocks for temporal feature extraction, followed by dense layers for classification:

- Conv1D → MaxPooling1D → Conv1D → MaxPooling1D → Flatten → Dense → Dropout → Output
- Activation functions: ReLU for hidden layers, Sigmoid for binary classification, Softmax for multiclass classification.
- Optimizer: Adam (learning rate = 0.0001).
- Loss functions:
  ○ Binary Cross-Entropy for tampered vs. original.
  ○ Categorical Cross-Entropy for multiclass classification.

Table 1 summarizes the structure of the proposed 1D-CNN for temporal tampering detection. The network processes a padded temporal sequence of five normalized forensic features extracted from consecutive frames. Two convolutional blocks capture local and higher-order temporal patterns, followed by flattening and dense layers for classification. Dropout regularization mitigates overfitting, and a sigmoid output provides tampering probability.

### 2.6. Dataset and Creating a Custom Dataset

The D^2^-City dataset is a large-scale collection of dashcam videos captured under diverse road, weather, and traffic conditions. It contains over 10,000 clips, each 30 s long at 25 frames per second, with resolutions of 720 p or 1080 p and bitrates ranging from 2000 to 4000 kbps. To preserve data diversity, original resolutions and bitrates were retained where applicable [37].

From this we selected a total of 1231 original videos (444 for deletion scenario, 408 for insertion scenario, and 379 for duplication scenario). Each selected original video was split into three non-overlapping 10-s clips, yielding 1231 × 3 = 3693 clips. To introduce temporal distortions in video sequences, three operations were applied: frame deletion, frame insertion, and frame duplication. For each operation, both the position within the video and the number of frames were randomly selected to ensure variability. Specifically, a sequence of 13 to 25 consecutive frames was either removed, inserted, or duplicated from one location and placed at another randomly chosen position. All details of these manipulations, including the selected positions and frame counts, were systematically recorded in an Excel file to guarantee reproducibility and support subsequent analysis.

The minimum threshold of 13 frames for deletion, insertion, or duplication was established based on human visual response characteristics. Empirical studies indicate that visual reaction times typically range between 200 and 250 milliseconds, after which an initial physical response may occur. However, executing purposeful motor actions, such as reaching, pushing, or adjusting body posture, generally requires a minimum of approximately 600 milliseconds [38,39]. Therefore, 13 frames, corresponding to about 0.52 s at 25 frames per second, cannot represent a complete and meaningful action. For this reason, a 13-frame manipulation was set in constructing the tampered dataset. All manipulations were performed using FFmpeg **version**: 8.0-full_build-www.gyan.dev and custom Python scripts; the exact generation code and the seed used for random locations are provided.

### 2.7. Software and Hardware Environment

All experiments were conducted using Python 3.12.10 with OpenCV, NumPy version: 2.2.6, scikit-learn version 1.7.2, TensorFlow 2.20.0, and Keras version 3.12.0. The hardware configuration included an Intel^®^ Core™ i7 processor (Intel, Santa Clara, CA, USA), 8 GB RAM, and Windows 10 operating system. No GPU was used; all training and inference were performed on CPU. This setup demonstrates the computational efficiency of the proposed model and its suitability for deployment in resource-constrained environments without dedicated GPU hardware.

## 3. Results

### 3.1. Detecting Frame Deletion Tampering

We used a subset of the D^2^-City dataset comprising 1323 videos, each 10 s long at 25 frames per second. For each original video, a corresponding tampered version was generated by randomly deleting 13–25 consecutive frames. After feature extraction, the model was trained using the derived feature vectors. We allocated 15% of the videos for testing and used the remaining 85% for training and validation (80% training, 20% validation).

**Figure 6:**
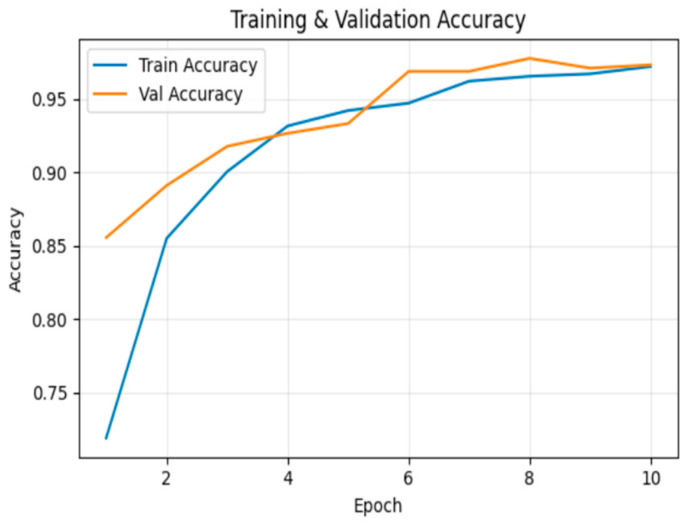
Training and validation Accuracy for frame deletion tampering over epochs.

Figure 6 illustrates the training and validation accuracy across epochs. By the 10th epoch, training accuracy reached 99.7%, while validation accuracy was 93.9%.

**Figure 7:**
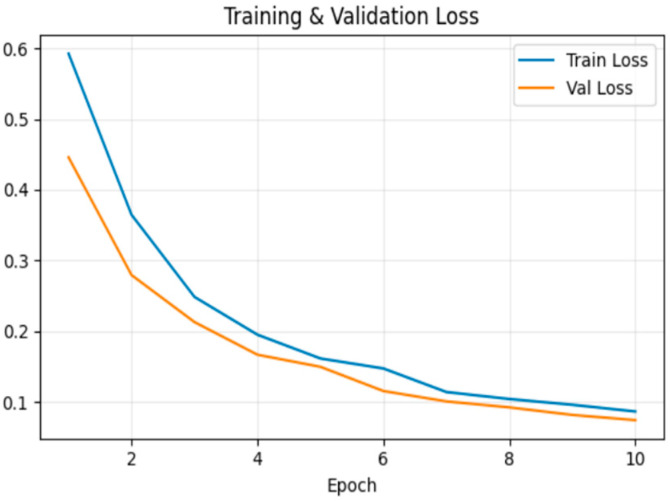
Model loss for frame deletion tampering over epochs.

Figure 7 shows the loss trend, which decreased steadily during training. At the end of the 10th epoch, training loss was 0.086 and validation loss was 0.074.

**Figure 8:**
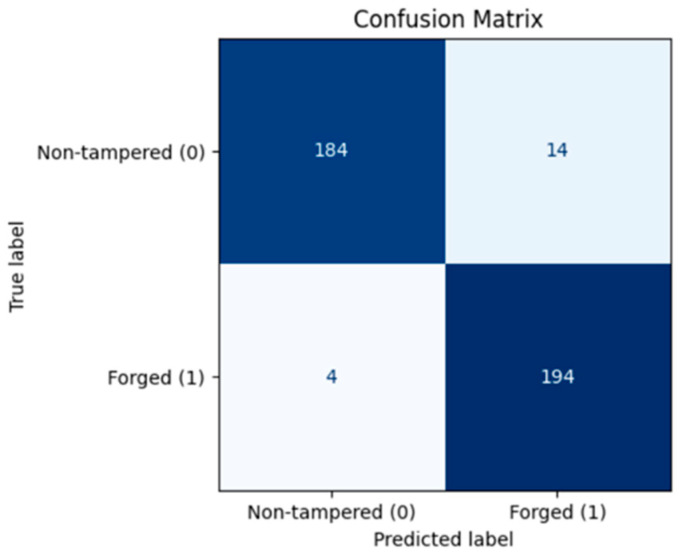
Confusion Matrix—Frame Deletion Tampering.

**Figure 9:**
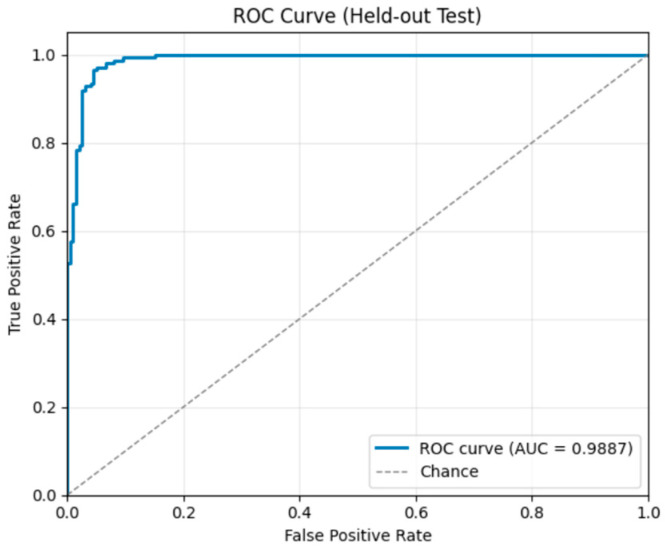
ROC Curve—Frame Deletion Tampering.

On the test set, the model achieved 95.0% accuracy, 96.0% precision, 95.0% recall, and an F1-score of 95.0%. The confusion matrix (Figure 8) indicates that, among 396 samples, 184 non-tampered videos were correctly classified, while 14 were misclassified as tampered. Similarly, 194 tampered videos were correctly identified, with only 4 misclassified as non-tampered, reflecting a very low error rate. The ROC curve (Figure 9) shows an AUC of 0.988, indicating excellent discrimination between original and tampered videos.

### 3.2. Detecting Frame Insertion Tampering

We used a subset of the D^2^-City dataset containing 1213 videos, each 10 s long at 25 frames per second. For each original video, a tampered version was generated by randomly inserting 13–25 consecutive frames at a random position. After feature extraction, the model was trained using the resulting feature vectors. We allocated 15% of the videos for testing and used the remaining 85% for training and validation (80% training, 20% validation).

**Figure 10:**
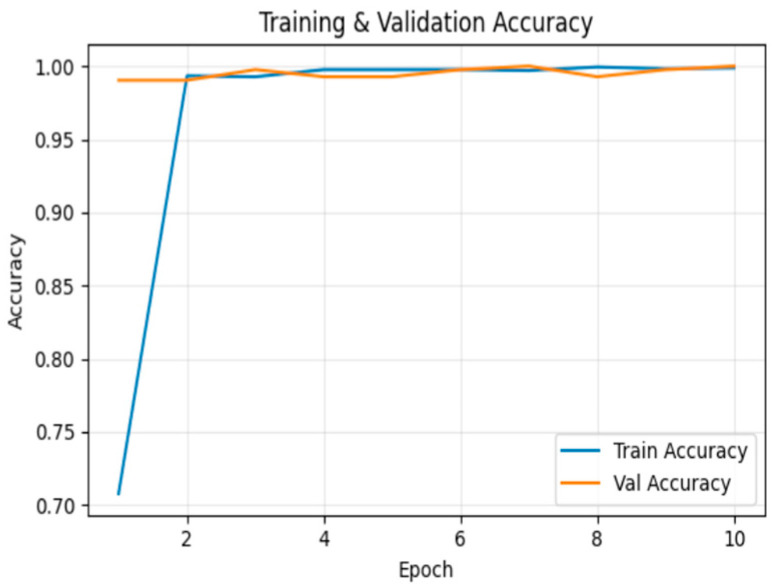
Training and validation accuracy for frame insertion tampering over epochs.

Figure 10 illustrates the training and validation accuracy across epochs. By the 10th epoch, training accuracy reached 99.8%, and validation accuracy was 100.0%.

**Figure 11:**
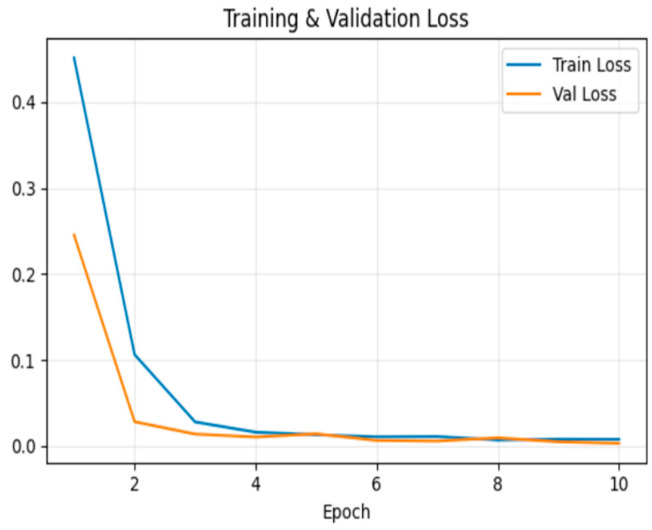
Model loss for frame insertion tampering over epochs.

Figure 11 shows the loss trend, which decreased steadily during training. At the end of the 10th epoch, training loss was 0.007 and validation loss was 0.003.

**Figure 12:**
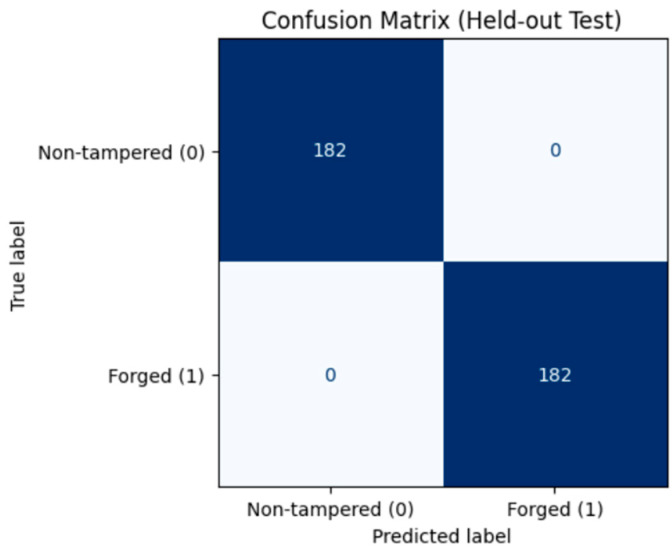
Confusion Matrix – Frame Insertion Tampering.

**Figure 13:**
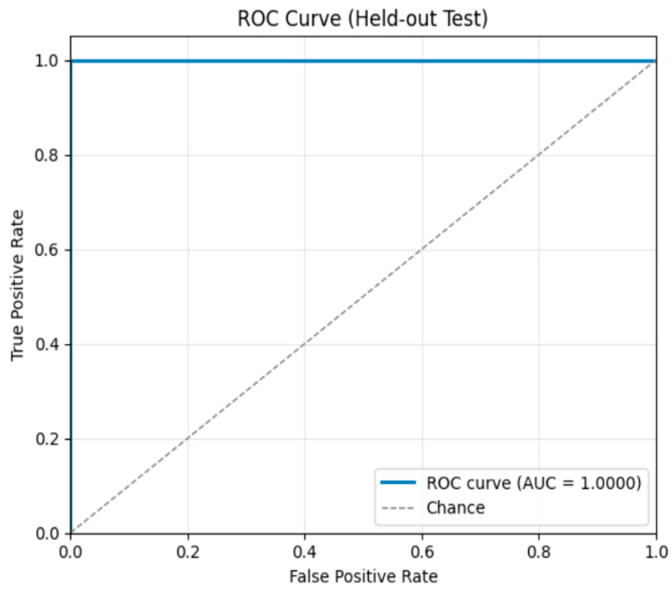
ROC Curve – Frame Insertion Tampering.

On the test set, the model achieved perfect performance: 100.0% accuracy, precision, recall, and F1-score. The confusion matrix (Figure 12) confirms that all 182 non-tampered and 182 tampered videos were correctly classified, with zero misclassifications. The ROC curve (Figure 13) shows an AUC of 1.0, indicating flawless discrimination between original and tampered videos.

### 3.3. Detecting Frame Duplication Tampering

We used a subset of the D^2^-City dataset containing 1065 videos, each 10 s long at 25 frames per second. For each original video, a tampered version was generated by duplicating 13–25 consecutive frames and inserting them at a random position. Both the source segment and insertion point were selected randomly. After feature extraction, the model was trained using the derived multi-feature vectors. We allocated 15% of the videos for testing and used the remaining 85% for training and validation (80% training, 20% validation).

**Figure 14:**
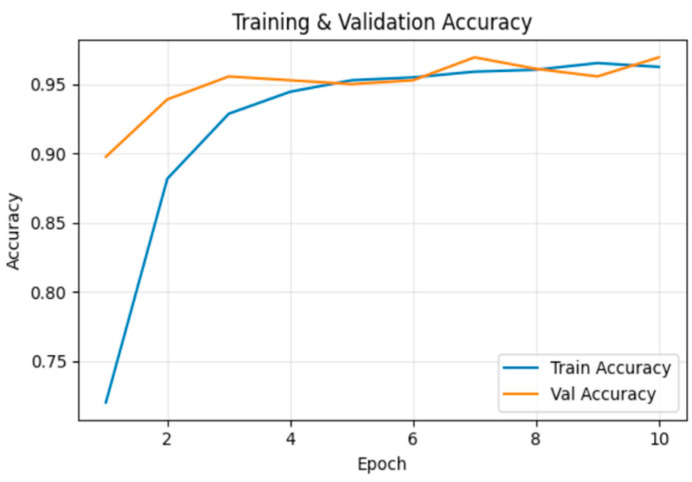
Training and validation accuracy for frame duplication tampering over epochs.

Figure 14 illustrates the training and validation accuracy across epochs. By the 10th epoch, training accuracy reached 96.2%, and validation accuracy was 96.9%.

**Figure 15:**
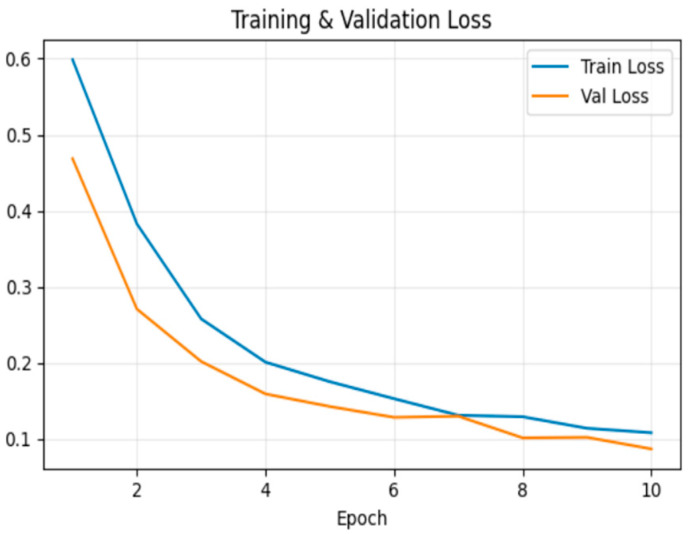
Model loss for frame duplication tampering over epochs.

Figure 15 shows the loss trend, which decreased steadily during training. At the end of the 10th epoch, training loss was 0.108 and validation loss was 0.087.

**Figure 16:**
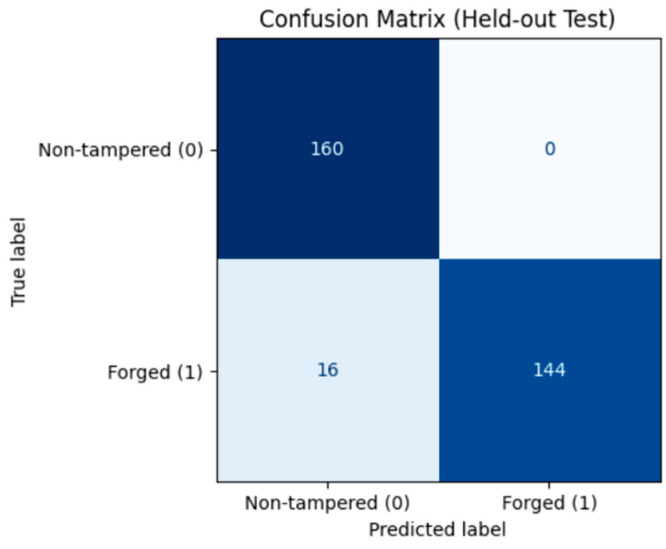
Confusion matrix—Frame Duplication Tampering.

**Figure 17:**
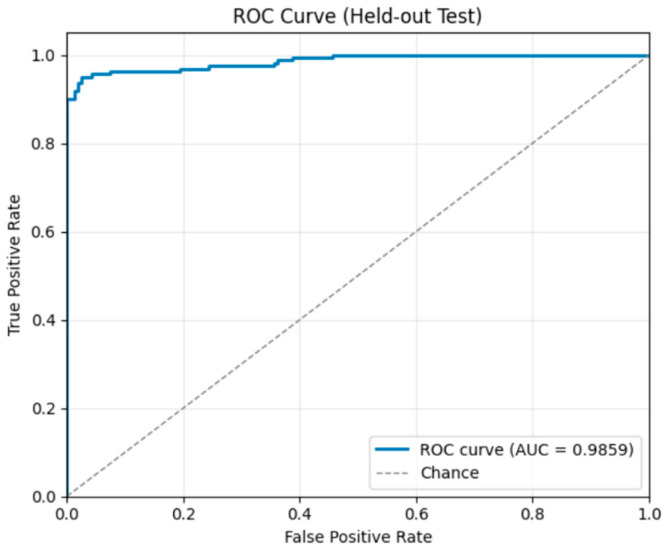
ROC curve—Frame Duplication Tampering.

On the test set, the model achieved 95.0% accuracy, 95.0% precision, 95.0% recall, and an F1-score of 95.0%. The confusion matrix (Figure 16) indicates that 160 non-tampered videos and 144 tampered videos were correctly classified, with only 16 tampered videos misclassified as non-tampered. The ROC curve (Figure 17) shows an AUC of 0.985, confirming strong sensitivity and low false positive rates, and demonstrating robust generalization on unseen duplication scenarios.

### 3.4. Multiclass Classification

To classify non-tampered videos and tampered videos involving frame insertion, deletion, and duplication, we trained a multiclass classification model. The dataset comprised 1323 videos with frame deletion, 1213 with frame insertion, 1065 with frame duplication, and 3601 non-tampered videos. We allocated 15% of the videos for testing and used the remaining 85% for training and validation (80% training, 20% validation).

**Figure 18:**
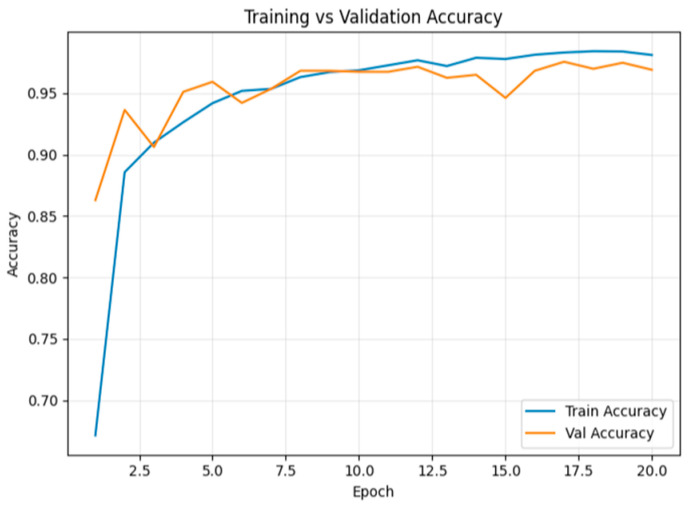
Training and validation accuracy for multiclass classification tampering over epochs.

Figure 18 illustrates the training and validation accuracy across epochs. By the 20th epoch, training accuracy reached 98.1%, while validation accuracy was 96.9%.

**Figure 19:**
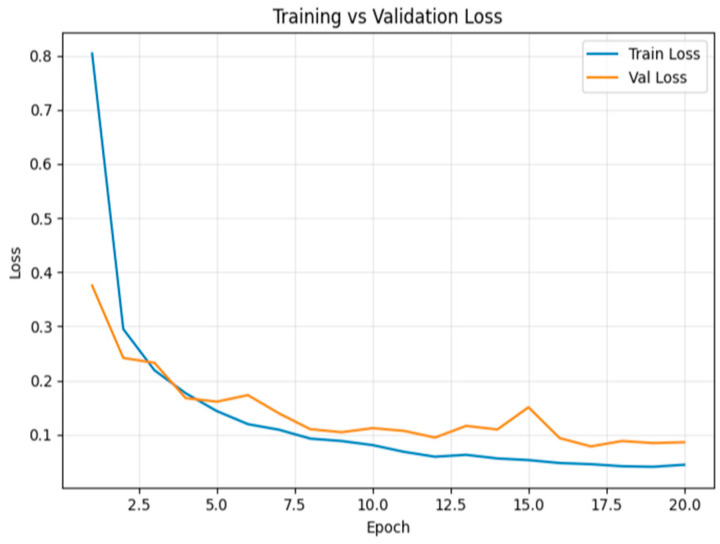
Model loss for multiclass classification tampering over epochs.

Figure 19 shows the loss trend, which decreased steadily during training. At the end of the 20th epoch, training loss was 0.044 and validation loss was 0.085.

**Figure 20:**
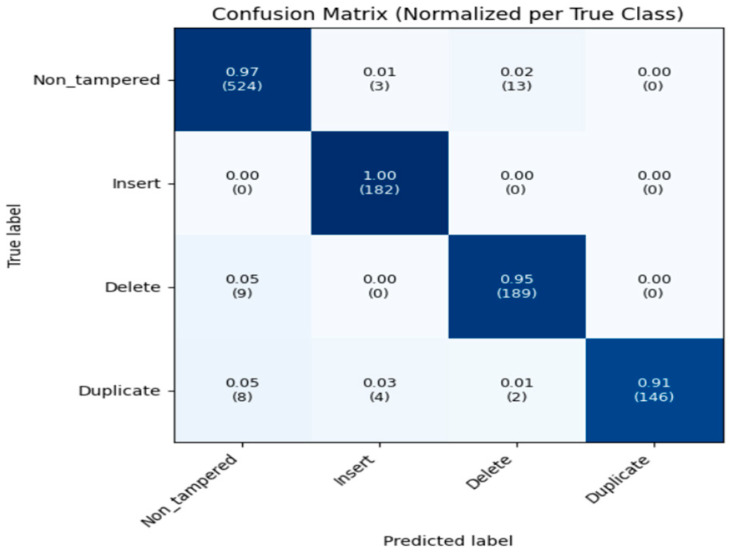
Confusion Matrix Multiclass Classification.

**Figure 21:**
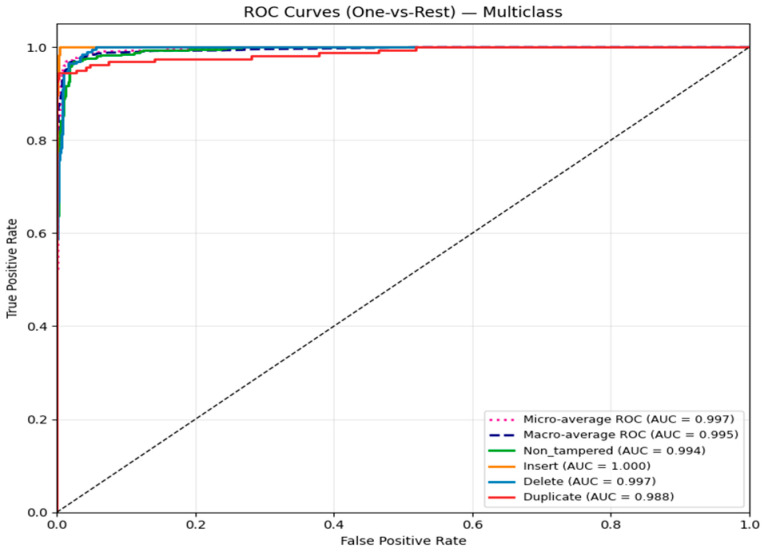
ROC curve for multiclass classification.

On the test set, the model achieved 96.3% accuracy, 96.4% precision, 95.9% recall, and an F1-score of 96.1%. The confusion matrix (Figure 20) shows that most predictions fall along the diagonal, indicating high accuracy: 524 non-tampered videos, 182 insertion cases, 189 deletion cases, and 146 duplication cases were correctly classified, with minimal misclassifications primarily between similar tampering types. The ROC curves (Figure 21) demonstrate excellent separability, with AUC scores of 0.994 for non-tampered, 1.0 for insertion, 0.997 for deletion, and 0.988 for duplication, confirming near-perfect classification performance across all classes.

### 3.5. Cross Dataset Experimentations

To assess both in-domain performance and cross-domain generalization, we first trained and tested the proposed model on the D^2^-City dataset. On the held-out subset, the model achieved near-perfect results: frame deletion, 95.0% accuracy, 96.0% precision, 95.0% recall, and 95.0% F1-score; frame insertion, 100.0% across all metrics; and frame duplication, 95.0% accuracy, precision, recall, and F1-score. These results confirm strong performance under similar conditions.

Cross-dataset evaluation revealed significant performance degradation. On VIRAT [40], frame insertion remained robust (≈97% accuracy, ≈99% precision, ≈95% recall), while frame deletion and duplication dropped to ≈50% accuracy, with forged-frame recall near zero, indicating severe domain shift. On BDDA [41], performance was intermediate: frame insertion achieved 81% accuracy (precision 0.90, recall 0.69), frame deletion fell to 53% accuracy (forged recall only 0.12), and frame duplication reached 51% accuracy but with forged recall as high as 0.94, suggesting a strong bias toward tampered detection. These results underscore the sensitivity of temporal tampering detection to dataset-specific motion patterns and camera perspectives, highlighting the need for domain adaptation and manipulation-aware augmentation to improve robustness.

### 3.6. Ablation Study

We conducted an ablation study to evaluate the impact of architectural design choices on the performance of the proposed 1D-CNN tampering detector. Three hyperparameters were varied: convolutional kernel size {3, 5, 7}, number of Conv1D blocks {1, 2}, and dropout rate {0.3, 0.5}. Each configuration was trained with three random seeds (42, 123, 2024) using the same dataset splits as the frame-deletion setting (Train: 1800 videos; Val: 450; Test: 396; input shape: (249, 5)). This resulted in 12 configurations (3 kernels × 2 depths × 2 dropouts), and performance metrics are reported as mean ± standard deviation across seeds.

Results indicate that network depth is the most influential factor. Two Conv1D + Pooling blocks consistently outperform single-block models: the best single-block configuration (kernel = 3, dropout = 0.5) achieved F1 = 0.9585 ± 0.0014, while the best two-block configuration (kernel = 7, dropout = 0.3) reached F1 = 0.9645 ± 0.0035. Kernel size also plays a significant role; larger kernels (5 and 7) improve temporal context modeling, with F1 increasing from 0.9608 ± 0.0046 (k = 3, two blocks, dropout = 0.5) to 0.9634 ± 0.0039 (k = 5, two blocks, dropout = 0.5) and peaking at 0.9645 ± 0.0035 (k = 7, two blocks, dropout = 0.3). Dropout rate has only a modest effect, with differences ≤ 0.004 F1, though configurations with dropout = 0.3 slightly outperform those with 0.5 for larger kernels.

Efficiency remained stable across all variants, with inference times clustered around 0.050–0.054 s per video. The best configuration for frame deletion used kernel = 7, two Conv1D blocks, and dropout = 0.3, achieving Test Accuracy = 0.9638 ± 0.0039 and Test F1 = 0.9645 ± 0.0035.

To assess robustness, we computed approximate 95% confidence intervals (CIs) for mean F1 scores. For example, the best-performing configuration (kernel = 7, two blocks, dropout = 0.3) achieved an F1 score of 0.9645 ± 0.0035, corresponding to a CI of [0.9558, 0.9732]. Although deeper architectures and larger kernels consistently yield higher performance, overlapping CIs suggest that improvements may not be statistically significant at the 95% level. Overall, depth and kernel size exert meaningful influence on temporal modeling, while dropout variations have minimal impact, see Table 2.

### 3.7. Efficiency Analysis

We assessed the runtime and memory efficiency of the proposed 1D-CNN framework across three experimental configurations: frame deletion, frame insertion, and frame duplication detection. Table 3 summarizes the results, which confirm that inference remains consistently lightweight across all scenarios.

The inference stage required only 0.0778–0.0791 s per video (≈12.71–12.87 FPS), demonstrating near real-time capability on standard CPU hardware. Preprocessing—comprising grayscale conversion, resizing, thresholding, and multi-feature extraction—dominated total runtime, averaging 3.65–4.07 s per video depending on tampering type. Memory overhead during inference was minimal (0.0847–0.0863 MB on average), with peak allocations ranging from 0.1392 MB to 0.7742 MB, confirming that the model remains computationally efficient and scalable even for complex manipulation patterns such as duplication.

These findings underscore the favorable trade-off between speed and resource usage offered by the proposed 1D-CNN compared to heavier temporal architectures (e.g., LSTM or 3D-CNN), which typically incur significantly higher inference times and memory demands [42,43]. In contrast, our approach provides a lightweight yet accurate solution, balancing strong detection performance with practical efficiency.

## 4. Discussion

The proposed multi-feature representation consistently captures localized anomalies across insertion, deletion, and duplication scenarios. These anomalies—plateaus for insertion, sharp spikes at splice points for deletion, and low intra-block motion with boundary discontinuities for duplication—enable reliable detection under per-video normalization. The 1D-CNN effectively learns these temporal patterns, achieving 95–100% accuracy in single-attack settings and 96.3% in the multiclass setting on D^2^-City. From a forensic perspective, the features are interpretable (e.g., SSIM drift for structural changes, optical flow cues for motion continuity), supporting explainable decisions and evidentiary reporting.

Compared to heavier temporal models such as 3D-CNNs or recurrent architectures, the proposed 1D-CNN combined with multi-feature analysis offers near real-time CPU inference (~0.078 s per 10 s clip, ≈12.7–12.9 FPS) and minimal memory overhead (~0.085 MB), making it suitable for on-vehicle edge devices and offline forensic workflows without GPUs. Although preprocessing dominates runtime (≈3.65–4.07 s per video), this stage remains tractable and can be parallelized or hardware-accelerated.

Recent surveys, such as [44], highlight that most interframe forgery detection methods are evaluated on surveillance or static-scene datasets (e.g., SULFA, LASIESTA, VIFFD, VTL) and often employ GPU-intensive architectures like 3D-CNNs or CNN-LSTM hybrids. These baselines differ fundamentally from our target domain—automotive dashcam videos—which exhibit strong egomotion, diverse lighting/weather conditions, and compression profiles. Direct numerical comparison would therefore be misleading without re-training all models on a unified dashcam benchmark. In light of this, we position our approach as complexity-aware and deployment-oriented, reporting near real-time CPU inference (≈12.7–12.9 FPS) and minimal memory overhead (~0.085 MB), while analyzing domain-shift challenges through cross-dataset experiments. Future work will include complexity-normalized baselines once standardized dashcam benchmarks become available.

Prior handcrafted methods rely on correlation inconsistencies, entropy-coded frame statistics, motion vectors, PFOV, and frequency-domain cues but often assume stationary backgrounds or degrade under compression/noise [3,4,5,6,7,8,9,10,16,21,25]. Recent deep learning approaches (2D/3D CNNs, CNN-RNN hybrids, contrastive learning) improve accuracy but are computationally expensive and typically evaluated on surveillance datasets [11,12,13,14,15,18,24,26,27,28]. Our results on dashcam data address these gaps by combining interpretable temporal features with a lightweight classifier, achieving strong accuracy and practical efficiency.

Cross-dataset experiments reveal sensitivity to camera placement, motion regimes, and scene semantics. Insertion remains relatively robust (≈97% accuracy on VIRAT; 81% on BDDA), while deletion and duplication degrade significantly (≈50–53% accuracy, with forged recall near zero for deletion on VIRAT and 0.12 on BDDA, and a bias toward tampered detection for duplication on BDDA). These findings suggest that distributional differences—such as egomotion strength, compression profiles, lighting/weather, and frame rate/resolution—affect feature statistics and learned temporal priors. Promising remedies include domain adaptation (e.g., feature-wise moment alignment, adversarial domain confusion), manipulation-aware augmentation (varying block length/position, re-encoding at diverse bitrates/codecs), and calibration strategies (threshold tuning per domain).

The ablation study confirms that temporal receptive field and network depth are key drivers of accuracy. Configurations with two Conv1D + Pooling blocks consistently outperform single-block models, and larger kernels (5–7) improve temporal context modeling. Dropout variations (0.3–0.5) have only a modest effect (≤0.004 F1 difference). These gains incur negligible inference penalties, with processing times clustered around 0.050–0.054 s per video and a compact model size (~12 MB), ensuring deployment practicality.

Limitations include reliance on grayscale, fixed-resolution frames, which may be affected by extreme illumination changes, motion blur, or rolling-shutter artifacts. Dataset construction uses controlled manipulations (13–25 consecutive frames), which may not cover all adversarial strategies (e.g., sparse or irregular edits). Minor implementation variance (e.g., dropout rate) can complicate strict reproducibility; we mitigate this through per-video normalization, seed control, and open-source scripts. Future work should explore robustness to codec changes, recompression, variable frame rates, mixed tampering, and incorporate domain adaptation to bridge cross-dataset gaps.

## 5. Conclusions

This study presents a computationally efficient multi-feature temporal analysis framework combined with a lightweight 1D-CNN for detecting frame insertion, deletion, and duplication in automotive dashcam videos. By converting inter-frame relationships into compact temporal signals and applying per-video normalization, the proposed method achieves 95–100% accuracy in single-attack scenarios and 96.3% accuracy in multiclass detection on the D^2^-City dataset, while maintaining near real-time CPU inference and minimal memory usage. These characteristics make the approach practical for forensic pipelines and resource-constrained IoT/edge deployments.

Cross-dataset experiments highlight domain-shift challenges—particularly for deletion and duplication—underscoring the need for domain adaptation, manipulation-aware augmentation, and calibration strategies to improve generalization. Future work will focus on (i) extending the model to localize tampered segments, (ii) incorporating self-supervised pretraining on large unlabeled dashcam datasets, and (iii) exploring hybrid flow/geometry priors and codec-aware features to enhance robustness across diverse camera platforms and driving conditions.

## Figures and Tables

**Table 1:** Layer-Wise Structure and Parameter Details of the Proposed 1D-CNN Model.

Layer	Output Shape *	Description
Input	(T, 5)	Temporal feature sequence (T = padded length)
Conv1D (64, k = 5)	(T, 64)	Temporal feature extraction
MaxPooling1D (2)	(T/2, 64)	Temporal downsampling
Conv1D (128, k = 5)	(T/2, 128)	Higher-level temporal patterns
MaxPooling1D (2)	(T/4, 128)	Further downsampling
Flatten	(≈T/4 × 128)	Vectorization
Dense (128)	(128)	Classification embedding
Dropout (0.5)	–	Regularization
Output (Sigmoid)	(1)	Tampering probability

* Output Shape indicates the dimensionality of the data after each layer in the network. The first dimension represents the temporal sequence length (**T**), which corresponds to the number of frames after padding. In this case, the padded length is **T = 249**, derived from the maximum sequence length in the training set. As the data passes through convolution and pooling layers, this temporal dimension is progressively reduced—typically by a factor of two at each pooling stage—resulting in shapes such as **T/2**, **T/4**, and so on. For example, after the first pooling layer, the sequence length becomes approximately **124**, and after the second pooling layer, it becomes **62**; The second dimension in the output shape represents the number of feature channels or neurons at that stage. These channels increase as the network goes deeper, allowing the model to learn richer representations. For instance, an output shape of **(T, 64)** means the sequence length remains **T**, but each timestep now has 64 feature maps. Similarly, **(T/4, 128)** indicates that the sequence length has been reduced to one-fourth of its original size, and each timestep contains 128 feature maps. This progressive reduction in temporal length, combined with an increase in feature channels, enables the network to capture both local and global temporal patterns efficiently while maintaining computational feasibility.

**Table 2:** Summary of ablation configuration.

Kernel Size	Conv1D Blocks	Dropout	Test Accuracy (mean ± std)	Test F1-Score (mean ± std)	Avg. Inference Time (s)
**3**	1	0.3	0.9562 ± 0.0039	0.9569 ± 0.0039	0.0514
**3**	1	0.5	0.9579 ± 0.0015	0.9585 ± 0.0014	0.0516
**5**	1	0.3	0.9529 ± 0.0053	0.9541 ± 0.0047	0.0515
**5**	1	0.5	0.9495 ± 0.0000	0.9507 ± 0.0005	0.0500
**7**	1	0.3	0.9529 ± 0.0053	0.9539 ± 0.0046	0.0527
**7**	1	0.5	0.9520 ± 0.0051	0.9534 ± 0.0050	0.0520
**3**	2	0.3	0.9562 ± 0.0053	0.9570 ± 0.0049	0.0527
**3**	2	0.5	0.9604 ± 0.0053	0.9608 ± 0.0046	0.0526
**5**	2	0.3	0.9596 ± 0.0044	0.9600 ± 0.0042	0.0507
**5**	2	0.5	0.9630 ± 0.0039	0.9634 ± 0.0039	0.0519
**7**	2	0.3	0.9638 ± 0.0039	0.9645 ± 0.0035	0.0534
**7**	2	0.5	0.9630 ± 0.0029	0.9630 ± 0.0030	0.0541

**Table 3:** Computational efficiency across all experiments.

Tampering Type	Test Videos	Avg. Preprocessing Time (s)	Avg. Inference Time (s ± SD)	FPS	Avg. Memory Overhead (MB)	Peak Memory Increase (MB)
Frame Deletion	396	3.6495	0.0791 ± 0.0080	12.71	0.0863	0.7742
Frame Insertion	364	3.9364	0.0778 ± 0.0014	12.86	0.0847	0.1387
Frame Duplication	320	4.0731	0.0778 ± 0.0024	12.87	0.0848	0.1392

## Data Availability

Supplementary data, scripts, and videos supporting this study are openly available at Zenodo under the Creative Commons Attribution 4.0 license, DOI: https://doi.org/10.5281/zenodo.17696973. Further inquiries can be directed to the corresponding author.

## Supplementary Materials

The following supporting information can be downloaded at: https://www.mdpi.com/article/10.3390/s26020517/s1, Figure S1: Example frame difference plots for tampered and original videos; Table S1: Detailed ablation study results for kernel size, depth, and dropout configurations; Video S1: Demonstration of frame deletion tampering detection; Video S2: Demonstration of frame insertion tampering detection; Video S3: Demonstration of frame duplication tampering detection; Script S1: Jupyter notebook for multiclass tampering detection (1D CNN Multi Class Classification.ipynb); Script S2: Jupyter notebook for frame deletion detection (1D CNN_Frame Deletion Detection.ipynb); Script S3: Jupyter notebook for frame insertion detection (1D CNN_Frame Insertion Detection.ipynb); Script S4: Jupyter notebook for frame duplication detection (1D CNN_Frame Duplication Detection.ipynb); Script S5: Dataset generation script for tampered videos (video_frame_deletion_insertion_duplication_generator.ipynb); Data S1: Readme_data.txt and README.txt describing dataset structure and usage; Other: requirements.txt listing Python dependencies.

## Abbreviations

The following abbreviations are used in this manuscript:

CNN	Convolutional Neural Network
1D CNN	One-Dimensional Convolutional Neural Network
IoT	Internet of Things
ROC	Receiver Operating Characteristic
AUC	Area Under the Curve
FPS	Frames Per Second
PFOV	Pseudo Flow Orientation Variation
LBP	Local Binary Pattern
SIFT	Scale-Invariant Feature Transform
RANSAC	RANSAC: Random Sample Consensus
KPCA	Kernel Principal Component Analysis
GRU	Gated Recurrent Unit
LSTM	Long Short-Term Memory
DCT	Discrete Cosine Transform
IRB	Institutional Review Board
